# Supplementary material for: Dynamical trade-offs arise from antagonistic coevolution and decrease intraspecific diversity
Source: Nat Commun. 2017 Dec 12;8:2059. doi: 10.1038/s41467-017-01957-8 (PMC5727225; doi:10.1038/s41467-017-01957-8)
Supplement: Supplementary file 1 — Supplementary Information [file 41467_2017_1957_MOESM1_ESM.pdf]

Supplementary Note 1: Rationale of parameter choice

We choose biologically reasonable parameter combinations for our model (Supplementary Table 1), because not all parameter values can be measured experimentally. In our experiments, we didn't observe large death events in the prey population even without nutrition for a certain time period, as it is usually found in unicellular organisms. We defined the intrinsic death rate of prey d_x to be much smaller than the baseline birth rate b_x. Mutation rates of prey and predator, μ_x and μ_y, were chosen with respect to their population sizes such that two species evolve on a similar time scale. We chose the other parameters, r_c, p and d_y, to be able to recover classical predator-prey cycles in the simplest control setup of one prey and one predator and no evolution.

Supplementary Table 1: Model parameters

Parameter	Definition	Value
b_x	baseline grow rate of prey	1.0
d_x	intrinsic death rate of prey	0.1
r_c	resource competition coefficient	0.00005
μ_x	mutation rate of prey per division	0.0001
p	scaling coefficient of the predation rate	0.005
d_y	intrinsic death rate of predator	0.5
k_{max}	maximum reproduction efficiency ratio of predator	0.3 or 0.6

Supplementary Note 2: Normally distributed mutations

We have shown how diversity changes when the mutants of prey and predator are drawn from a uniform distribution. Here, we use an alternative choice of mutant distribution, i.e. the mutant trait is a random number from a normal distribution around its parent's trait (truncated to reflect the maximum trait ranges if necessary). In this scenario, mutants are on average more similar to their parents compared to a uniform distribution, especially when the variance of the normal distribution is small.

We investigate two representative cases, where the standard deviations of the normal distribution are large (100%) and small (10%) compared to the full trait range. When

the standard deviation is large, the mutants can move larger steps and thus we expect the dynamics to be more similar to those under a uniform distribution. This is exactly what we observed in our simulations, when the standard deviation is 1.0 for the prey and 0.3 for the predator (compare Supplementary Fig. 1 to Fig. 5 in the main text).

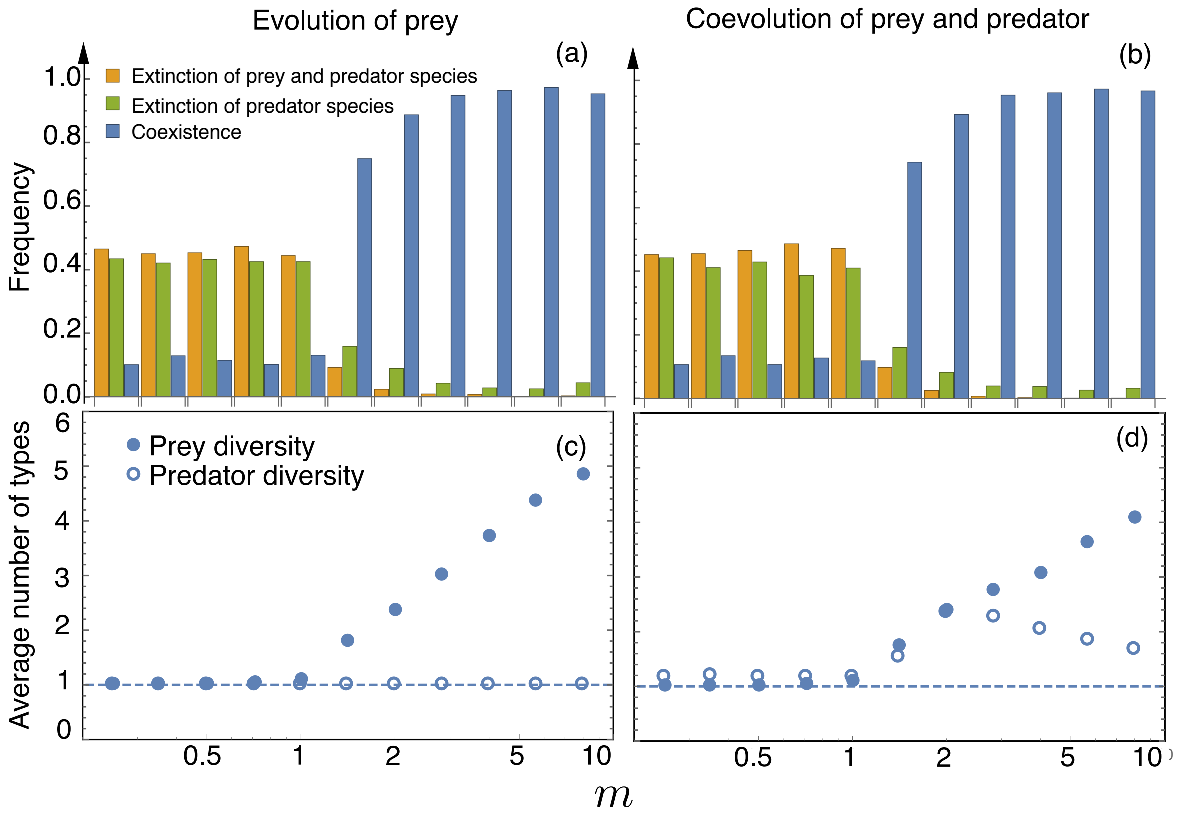

Supplementary Figure 1: **Diversity for coevolution and evolution under a normal mutant distribution with a large variance.** We observe a similar pattern of prey and predator diversity as well as the frequency of species extinction and coexistence compared to those under a uniform distribution. The standard deviations of prey and predator mutant are 1.0 amd 0.3 respectively (Other parameters: $b_x = 1.0$, $d_x = 0.1$, $\mu_x = 0.0001$, $r_c = 0.00005$, $d_y = 0.5$, $\mu_y = 0.001$, $p = 0.005$, $k_{\text{max}} = 0.3$, $k_1 = k_{\text{max}}$, $X_1(0) = 1000$, $Y_1(0) = 100$, $g_1 = 1.0$, averaged over 1000 independent runs and time period equals to 2000 per each run. The values for parameter m in the upper panels correspond to the points in the lower panels).

When the standard deviation is small, i.e., 0.1 for the prey and 0.03 for the predator, the general patterns of diversity differences between evolution and coevolution are again similar (Supplementary Fig. 2) to those under a uniform distribution (Fig. 5 in the

main text). That is when $m > 1$, prey diversity increases with m under both evolutionary and coevolutionary processes, and predator diversity increases first and decreases again under coevolution. Prey diversity is higher under only prey evolution than under coevolution. However, we observe quantitive differences. First, we see a higher frequency of the prey extinction which leads to the extinction of both species (orange bars in Supplementary Fig. 2). Second, under the coexistence of both species, we see a slightly higher diversity for both prey and predator compared to those under a uniform distribution (Fig. 5 in the main text) or a normal distribution with a larger variance (Supplementary Fig. 1). As the mutants move smaller steps under a normal distribution with a small variance, it is more difficult for species to evolve away from their current states, either facing extinction or coexistence of multiple types.

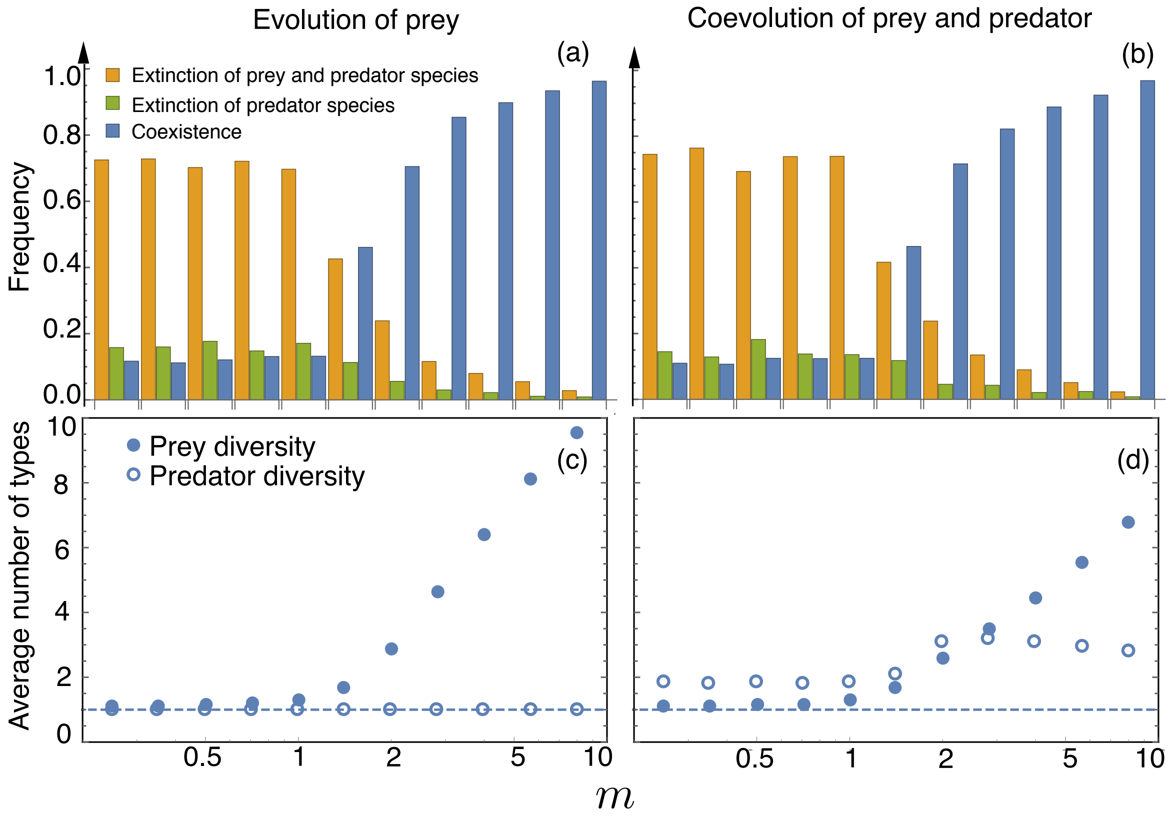

Supplementary Figure 2: **Diversity for coevolution and evolution under a normal mutant distribution with a small variance.** The standard deviations of prey and predator mutant are 0.1 amd 0.03 respectively. We observe a higher frequency of extinction of both prey and predator compared to a uniform distribution or a normal distribution with a larger variance. However, the diversity differences between evolution and coevolution are similar and independent of the choice of mutant trait distributions. (Other parameters: $b_x = 1.0$, $d_x = 0.1$, $\mu_x = 0.0001$, $r_c = 0.00005$, $\mu_y = 0.001$, $p = 0.005$, $k_{\text{max}} = 0.3$, $k_1 = k_{\text{max}}$, $X_1(0) = 1000$, $Y_1(0) = 100$, $g_1 = 1.0$, averaged over 1000 independent runs and time period equals to 2000 per each run. The values for parameter m in the upper panels correspond to the points in the lower panels).

Supplementary Note 3: The impact of k_{max}

We have defined k as the ratio of predator growth to the predation ability. Mutations lead to predator types with new k values. The maximum value, k_{max}, represents the constraint of the predator species transferring resource to offspring. Since a predator must consume to reproduce, we used a small $k_{\text{max}} = 0.3$ in our main text. The predator can potentially produce more offspring when consuming the same amount of prey under larger k_{max} values. This leads to a higher frequency of prey extinction and thus ultimately the extinction of both species (orange bars in Supplementary Fig. 3a&b). Especially when $m < 1$, where extinction is already likely for $k_{\text{max}} = 0.3$ (Fig. 5 in the main text), we do not observe any coexistence of prey and predator species when $k_{\text{max}} = 0.6$ (blue bars in Supplementary Fig. 3a&b).

Interestingly, when the prey and predator coexist under $m > 1$, the diversity patterns under large k_{max} (Supplementary Fig. 3c&d)) is consistent with those under small k_{max}. Prey diversity increases with m for both evolution of prey and coevolution, whereas predator diversity increases first and then decreases with m under coevolution. Prey diversity is higher under evolution of prey than coevolution of prey and predator. Thus, k_{max} does not change the diversity differences between evolution and coevolutionary process when both species coexist.

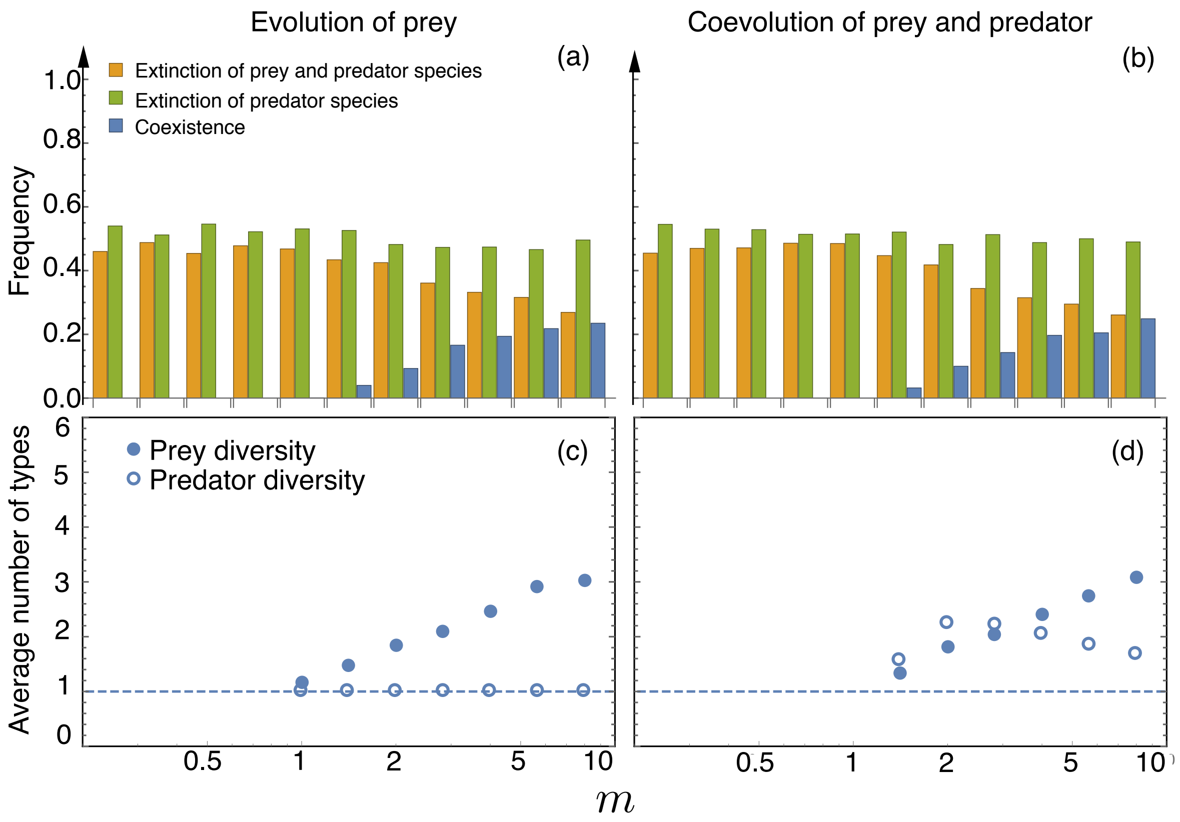

Supplementary Figure 3: **Coevolutionary and evolutionary diversity under $k_{\text{max}} = 0.6$.** The general pattern we found for lower values of k_{max} is recovered (Other parameters: $b_x = 1.0$, $d_x = 0.1$, $\mu_x = 0.0001$, $r_c = 0.00005$, $d_y = 0.5$, $\mu_y = 0.001$, $p = 0.005$, $X_1(0) = 1000$, $Y_1(0) = 100$, $g_1 = 1.0$, $k_1 = k_{\text{max}}$, averaged over 1000 independent runs and time period equals to 2000 per each run. The values for parameter m in the upper panels correspond to the points in the lower panels).

Supplementary Note 4: Distinct prey types lead to out-of-phase cycles

Starting from the same homogenous populations with an ancestor prey and predator, we observe different predator-prey cycles in our stochastic simulations (see Supplementary Fig. 4 a&b). In classical predator-prey cycles, the peaks in total predator population size follow the peaks in total prey population size with a one-quarter phase shift; in out-of-phase cycles, the peaks in predator population size appear almost at the same time as the valleys in prey population. The left panels of Supplementary Fig. 4

show the dynamics under a relatively costly trade-off curve ($m = 3$). In this example, predator-prey abundances become out-of-phase cycles for a short period when two distinct prey types coexist in the population (time 50 to 200), and return to be classical cycles again when there is only one type dominating the population. When the initial trade-off curve is very cheap, e.g $m = 10$, we observe more coexisting types and out-of-phase cycles are more prevalent (Supplementary Fig. 4b).

We record the prey growth (g values) and predation capability for predators (negatively correlated to k values) as well as their frequencies over time. Under a relatively costly trade-off curve ($m = 3$), prey individuals have different growth which refers to relatively different defence level (Supplementary Fig. 4c). Meanwhile, predators with different predation capability are also present (Supplementary Fig. 4e). Under $m = 10$, while there is still a diversity of prey types, we observe only few predator types all with high predation capability (low k values) as all prey types have a relatively high defence (Supplementary Fig. 4d&e).

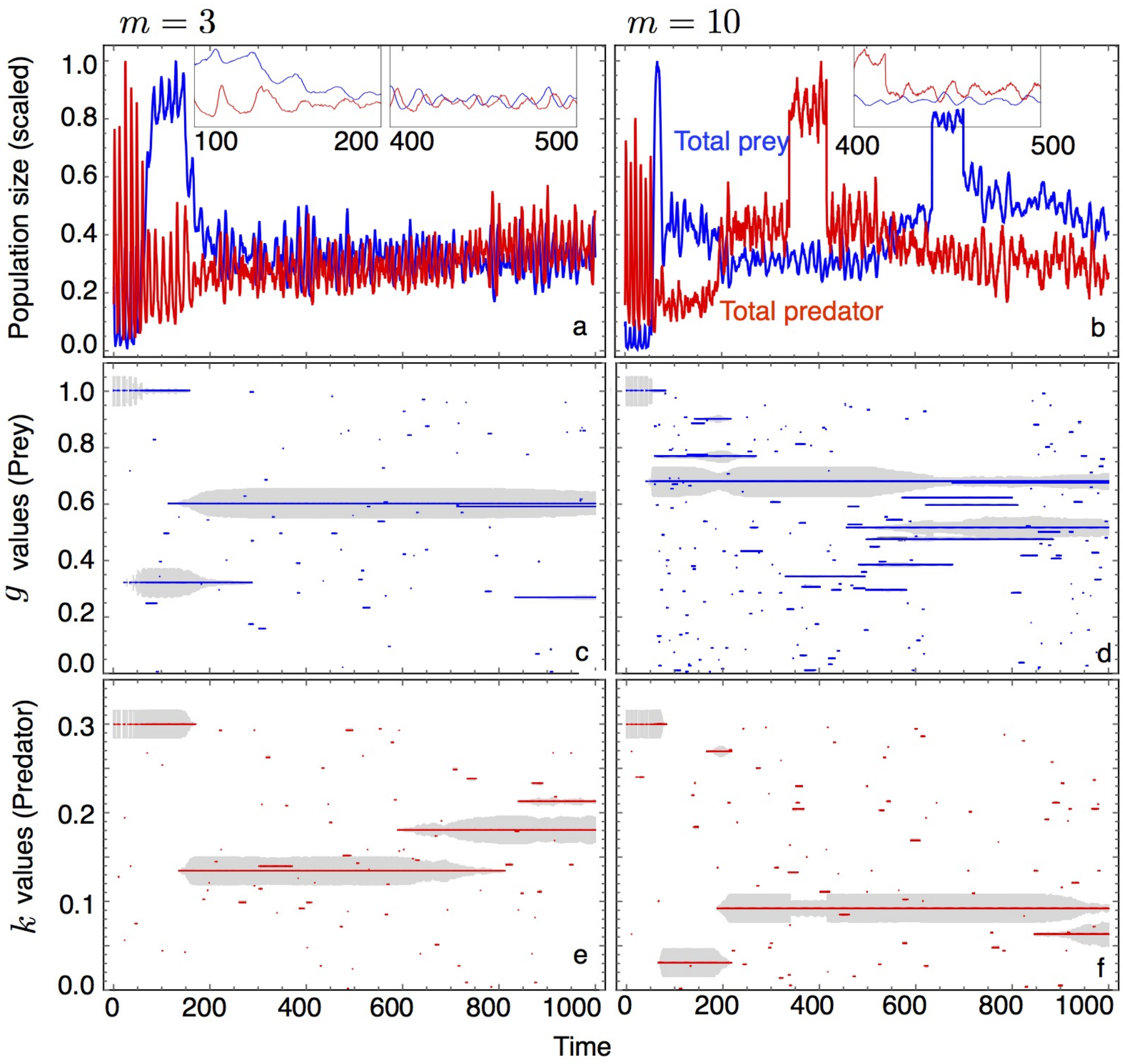

Supplementary Figure 4: **Population cycles under two different trade-off curves.** Starting from the same initial condition, we show examples where the system evolves to two different population cycles in the total prey and predator abundances. Left panels (m=3): classical predator-prey cycles are observed (a: inner panel between time 400 and 500) when only one prey type dominates the population(c); out-of-phase cycles (a: inner panel between time 100 and 200) when two prey types with different growth traits coexist. Right Panels (m=10): out-of-phase predator-prey cycles (b) are observed almost the whole time period as multiple prey types with different growth traits coexist (d). In the upper panels, the population sizes of prey and predator population are rescaled by their maximum values respectively. The lower panels show the g and k values of each prey and predator type. The width of the grey area represents the frequency of the corresponding type. The maximum frequency 1.0 has a width of 0.1 as the starting types (parameters $b_x = 1.0$, $d_x = 0.1$, $\mu_x = 0.0001$, $r_c = 0.00005$, $d_y = 0.5$, $\mu_y = 0.001$, $p = 0.005$, $X_1(0) = 1000$, $Y_1(0) = 100$, $g_1 = 1.0$, $k_1 = k_{\text{max}} = 0.3$).

Supplementary Note 5: Invasion analysis

We first calculate the fixed points of the predator-prey system in homogenous prey and predator populations, and further analysis the invasion of a new prey or a new predator with a different g (prey) or k (predator) value. To simplify our notation, we use g and k for an arbitrary prey and predator type, g_s and k_s for the equilibrium strategies. Based on the rate equations Eq. 4 in the main text in a monomorphic system, prey and predator abundances x and y change according to the deterministic equations

$$\dot{x} = x\left(b_x g - r_c x - d_x - y f\left(g,k\right)\right) = x \underbrace{\left(b_x g - r_c x - d_x - yp\, g^{\alpha k}\right)}_{\lambda_x} \tag{1}$$

and

$$\dot{y} = y\left(xkf\left(g,k\right) - d_y\right) = y \underbrace{\left(xkp\, g^{\alpha k} - d_y\right)}_{\lambda_y}, \tag{2}$$

where we introduced $\alpha = m/k_{\max}$. Thus, the non-trivial equilibrium of the prey population with $x, y > 0$ is given by

$$x^* = \frac{d_y}{kf\left(g,k\right)} = \frac{d_y}{kp} g^{-\alpha k}, \tag{3}$$

The non-trivial equilibrium of the predator population is

$$y^* = \frac{1}{f\left(g,k\right)}\left(b_x g - r_c x^* - d_x\right) = \frac{b_x}{p} g^{-\alpha k+1} - \frac{r_c d_y}{kp^2} g^{-2\alpha k} - \frac{d_x}{p} g^{-\alpha k}. \tag{4}$$

To assess whether a prey type with an alternative g can invade the population of prey and predator at equilibrium, we look at the direction of change of the prey growth rate in dependence of g at the fixed point. Based on Eq.1, this becomes

$$\left[\frac{\partial}{\partial g}\lambda_x\right]_{x^*,y^*} = b_x - y^*\, p\, \alpha k\, g^{(\alpha k-1)}. \tag{5}$$

Substituting the equilibria conditions Eq. 4, we have

$$\left[\frac{\partial}{\partial g}\lambda_x\right]_{x^*,y^*} = b_x\left(1-\alpha k\right) + \underbrace{\left(d_x k + \frac{r_c d_y}{p} g^{-\alpha k}\right)\frac{\alpha}{g}}_{>0}. \tag{6}$$

All parameters in the second term of Eq. 6 are positive. When $\alpha k < 1$, the first term is also positive and we have $\left[\frac{\partial}{\partial g}\lambda_x\right]_{x^*,y^*} > 0$. In this case, the prey population will evolve towards higher g if the predator doesn't change. By the definition of α in Eq. 3, this is equivalent to $mk < k_{\max}$. For a "naive" predator population, where the predation ability is the lowest ($k = k_{max}$), this invasion condition equals $m < 1$. However, if $\alpha k > 1$, the first term of Eq. 6 becomes negative and there will be interior equilibrium strategies, g_s, determined by the explicit solutions of $\left[\frac{\partial}{\partial k}\lambda_y\right]_{x^*,y^*} = 0$.

These invasion and equilibrium conditions of prey are consistent with our observation in simulations (Fig. 5 in the main text), where the average number of prey types is one when $m < 1$ and increases with m when $m > 1$. Especially when $m < 1$ where we observe mainly one prey and one predator type, the g values of the prey in most realisations evolve towards its maximum value at $g = 1$ (Supplementary Fig. 6).

The change of the predator growth at the fixed point, (x^*, y^*), can be obtained from Eq. (2) as

$$\left[\frac{\partial}{\partial k}\lambda_y\right]_{x^*,y^*} = x^* p g^{\alpha k}\left(1 + \alpha k \ln(g)\right). \tag{7}$$

Substituting the prey equilibrium from Eq. (3) leads to

$$\left[\frac{\partial}{\partial k}\lambda_y\right]_{x^*,y^*} = \left(\frac{1}{k} + \alpha \ln(g)\right) d_y. \tag{8}$$

The first term $1/k$ is always positive, whereas $\ln(g)$ is negative ($g \in [0,1]$). This implies a predator type with a higher k can invade if $k < -\frac{1}{\alpha \ln(g)}$ and a predator type with a lower k can invade if $k > -\frac{1}{\alpha \ln(g)}$ respectively. The equilibrium strategy k_s is given by

$$\left[\frac{\partial}{\partial k}\lambda_y\right]_{x^*,y^*} = 0 \iff \frac{1}{k^s} = -\alpha \ln(g) \tag{9}$$

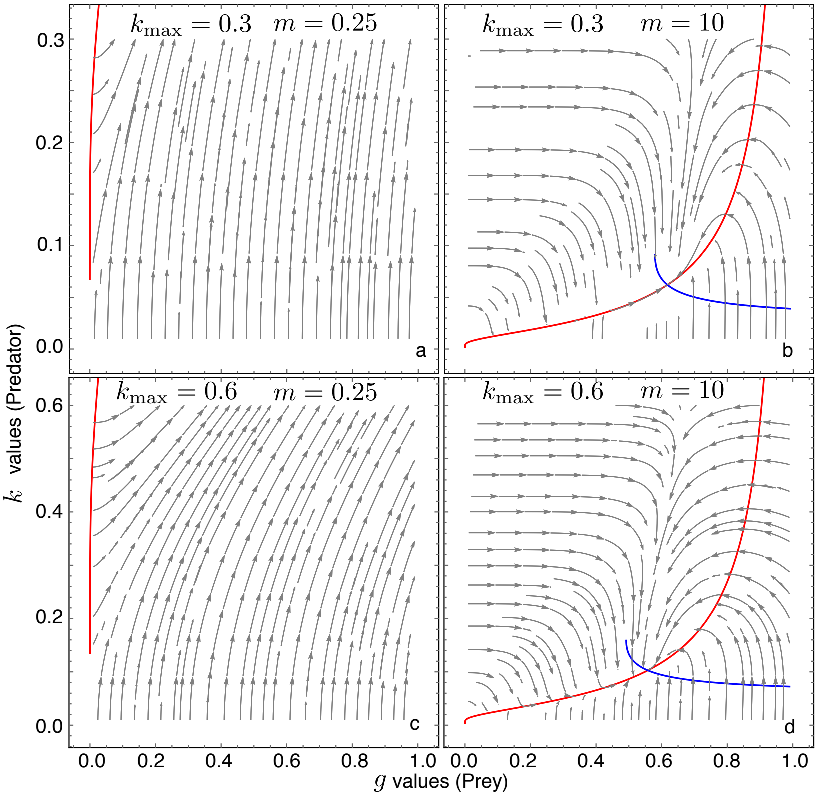

Supplementary Figure 5: **Phase portrait of the adaptive dynamics for Eqs.** (6) **and** (8) **in the Supplementary Note 1.** The grey lines with arrows show the trajectories of prey and predator traits. The blue line is the numerical equilibrium of Eq. (6) in the Supplementary Note 1 for the prey, and the red line of Eq. (9) in the Supplementary Note 1 for the predator. For a small m, both g and k evolve to their maximum value (panel a & c). When m is large, g and k will evolve to intermediate values. The value of k_{max} does not change the general pattern of the adaptive dynamics (all other parameters are the same as Fig. 5 of the main text, i.e. $b_x = 1.0$, $d_x = 0.1$, $r_c = 0.00005$, $d_y = 0.5$, $p = 0.005$).

For the stability of this predator strategy, we find

$$\left[\frac{\partial^2}{\partial k^2}\lambda_y\right]_{\frac{1}{k_s}=-\alpha\ln[g]} = -\frac{d_y}{k_s^2} = -d_y\alpha^2\ln^2(g) < 0 \tag{10}$$

Thus, the singular strategy is a fitness maximum and an evolutionary stable strategy if the prey type is fixed at (x^*, y^*). If $g = 1.0$, $k_s \to \infty$ according to Eq. (9) and the predator species will evolve to higher k if the prey does not change. Combining the invasion analysis of the prey and the predator, we expect that the predator and prey both evolve to their maximum values under $m < 1$ (Supplementary Fig. 5), which agrees with our observation in simulations (Supplementary Fig. 6).

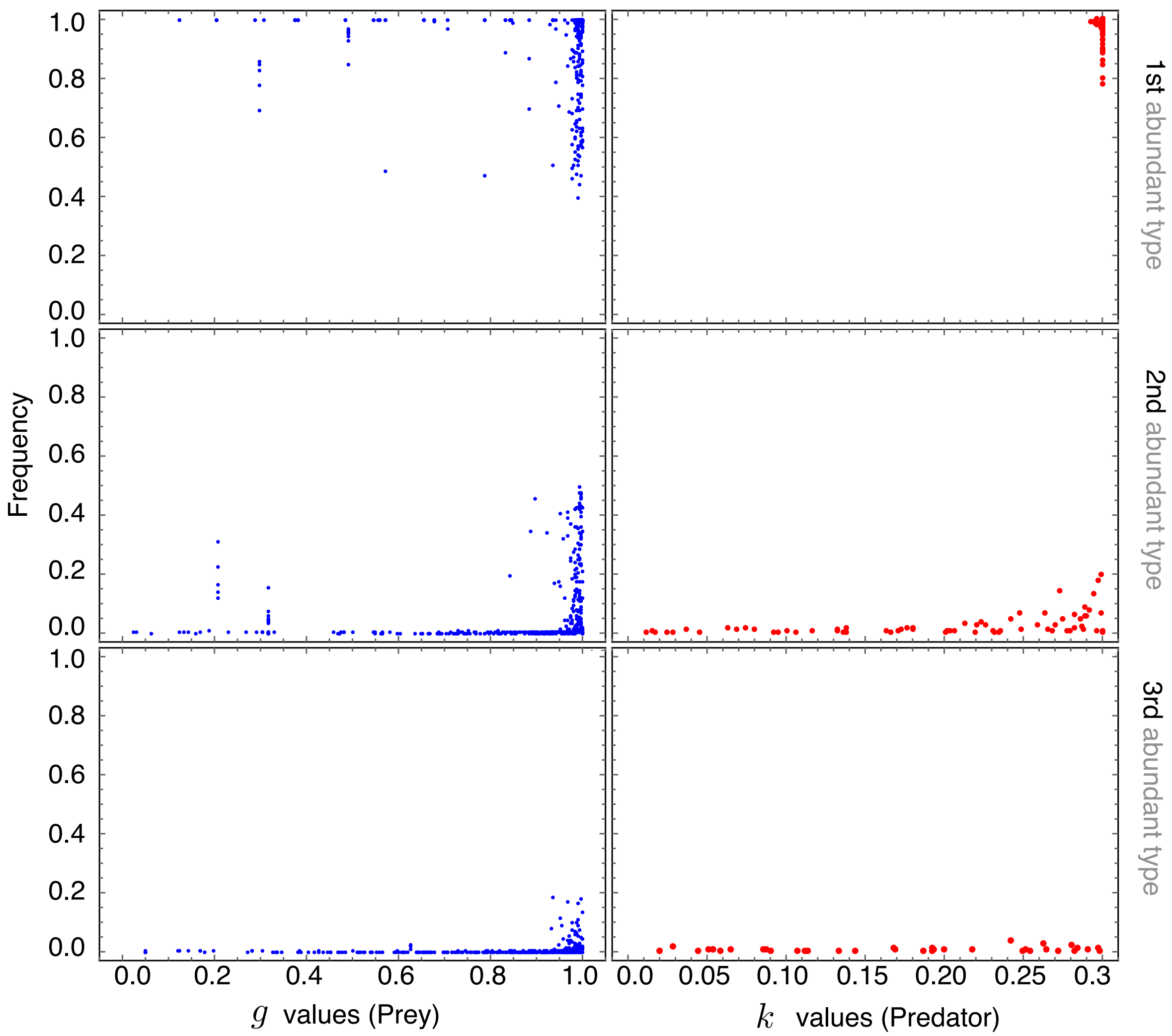

Supplementary Figure 6: **The three most abundant prey and predator types for $m = 0.25$.** Here we present an example figure of the g and k values of the most abundant types when defense is costly $m < 1$. The y-axis refers to the frequencies of corresponding types, each dot corresponds to a single realization. In total we show 1000 independent realisation of the same stochastic process. The upper panels show the most abundant types, the middle panels the second most abundant types, and the lower panels the third most abundant types. For most realisations, a single type with g close to 1.0 dominates the prey population and a single type with k close to k_{max} dominates the predator population (other parameters: all data points are obtained from the same simulations as in the Fig. 5 of the main text).

Supplementary Note 6: Quantifying differences among present types

Starting with an ancestor prey type ($g = 1$) and an ancestor predator type ($k = k_{max}$), we see that the prey diversity increases with m under the evolution of only prey and the coevolutionary process. In addition, under coevolution the predator diversity increases first and later decrease again with m. Here, we further explore the composition of the predator and prey populations and quantify diversities in terms of trait differences among present types. We measure the standard deviation of types in both prey and predator populations for each realisation.

For $m \leq 1$, we see a high probability to observe a standard deviation of 0 for both prey and predator types (Supplementary Fig. 7 and Supplementary Fig. 8). This is consistent with our observation that on average only a single type is maintained in prey and predator populations when defence is costly (Fig. 5c&d in the main text).

Although we observe a higher prey diversity for a given $m > 1$ under evolution compared to coevolution, the distributions of standard deviations of prey types are wider under coevolution, i.e. the differences between prey types are larger under coevolution (Supplementary Fig. 8) compared to evolution (Supplementary Fig. 7). While the background predation pressure remains constant without coevolution, the scenario complicates considerably under coevolution as multiple predator types with potentially large phenotypic differences exist (Supplementary Fig. 8). Coevolution of the predator does not only lead to a different prey diversity in terms of number of existing types, but also a different variance among those types (Supplementary Fig. 8).

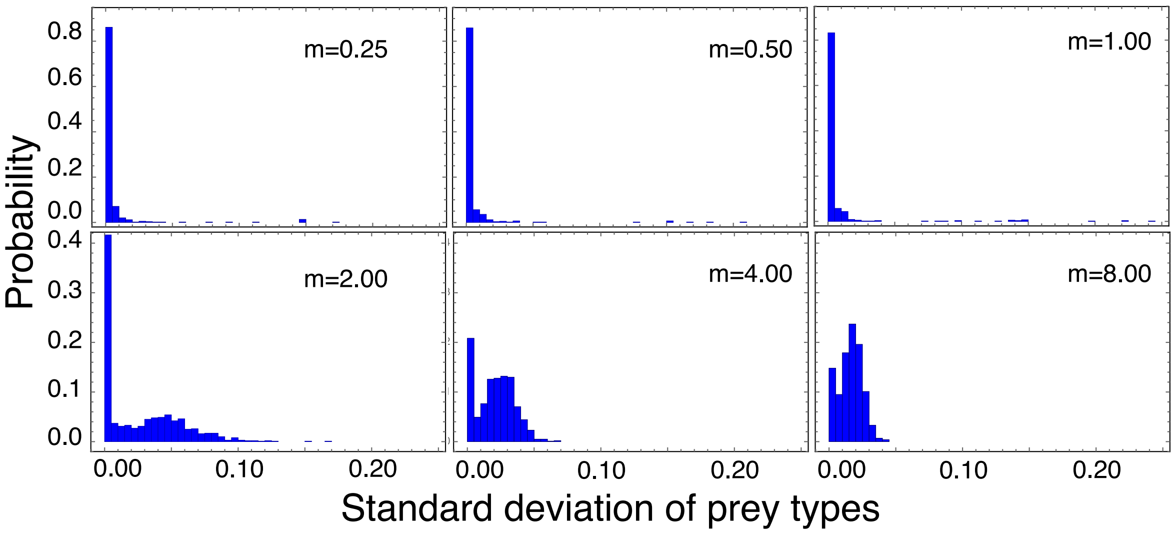

Supplementary Figure 7: **Distributions of standard deviations of prey types under the evolution of only prey.** Each panel refers to the distribution of standard deviations of present prey types in the end of 1000 realisations for a given m. The full range of the prey traits is $(0, 1]$. Note when there is only one type in the population, the standard deviation will be 0. When $m <= 1$, the average number of prey types is one (Fig. 5c in the main text). Correspondingly, we see a high probability of the standard deviation to be 0. (parameters: all data points are obtained from the same simulations as in the Fig. 5 of the main text).

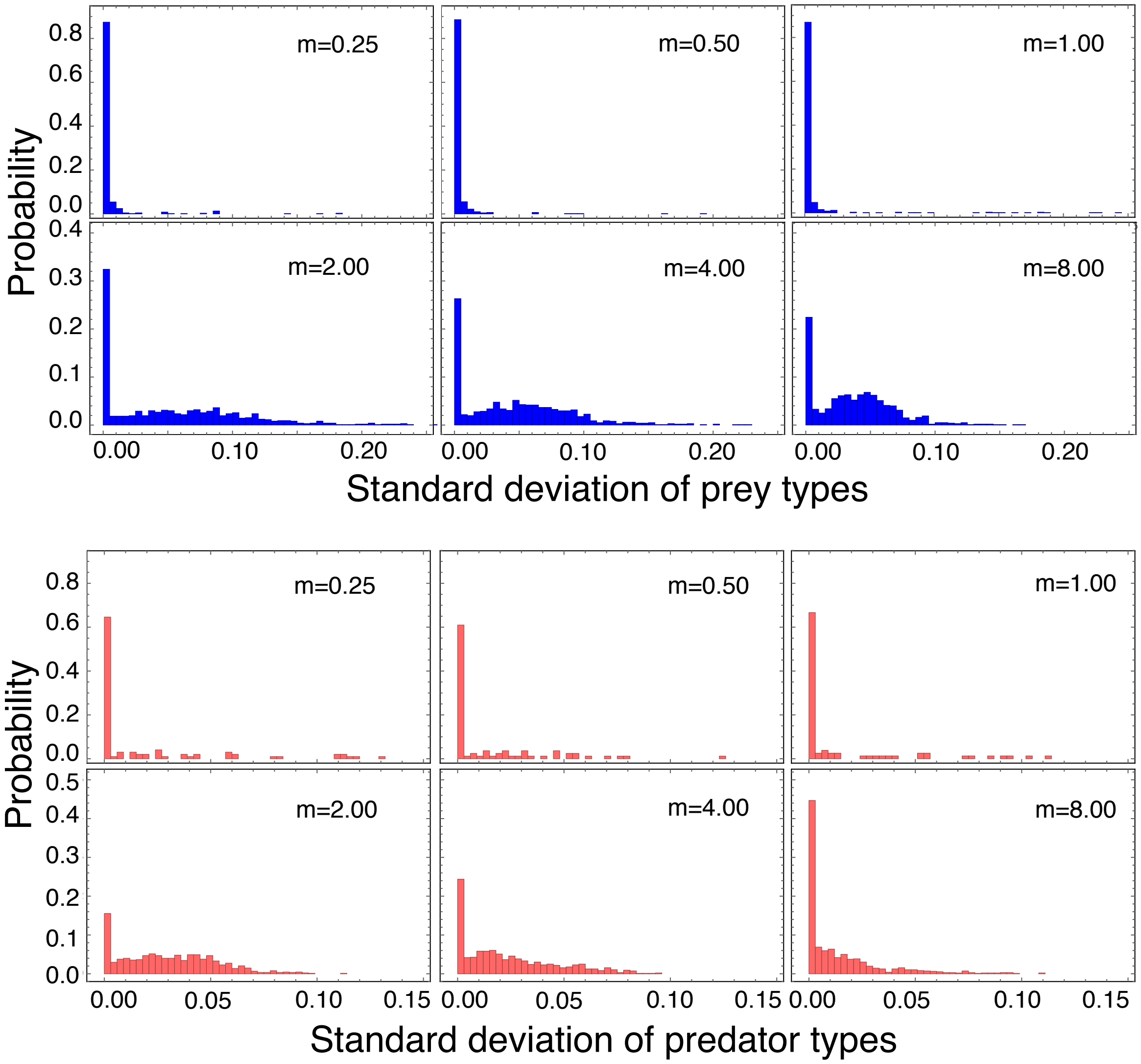

Supplementary Figure 8: **Distributions of standard deviations of prey and predator types under coevolution.** Each panel refers to the distribution of standard deviations of present prey or predator types at the end of 1000 realisations for a given m. When there is only one type in the population, the standard deviation will be 0. As prey can mutate in $(0, 1]$ and the predator in $(0, 0.3]$, the standard deviation of prey types can be up to 25% of the full range, and the standard deviation of predator types up to 40% of the full range. In general the variation of prey types is higher in a coevolutionary compared to a prey only evolution scenario (parameters: all data points are obtained from the same simulations as in the Fig. 5 of the main text).

Supplementary Note 7: Starting with evolved prey and predator types

Inspired by our experiment, we started simulations with the ancestor prey and predator types with the lowest defense and lowest predation ability. Here, we explore the impact of an opposite scenario starting with highly evolved types, i.e. small g and small k values compared to their maximum values. As the prey is highly defended (small g), the predator cannot consume the prey population rapidly. Even if the predator can consume prey, it spends little energy on reproduction (small k) and its population size increases only slowly. This leads to a high extinction rate of predator populations (see Supplementary Fig. 9a&b). However, if the predator escapes extinction and coexists with the prey, under coevolution both species can evolve to a similar diversity level (Supplementary Fig. 9d) as in the case starting from large g and k values (Fig. 5d in the main text).

Under the evolution of only prey, the predation pressure is alway high (small k) as the predator does not evolve. Thus, selection favours highly defended prey and the prey diversity is low even for relatively large m. Only if the defence is extremely cheap (e.g. $m > 5$), we observe an increase of prey diversity.

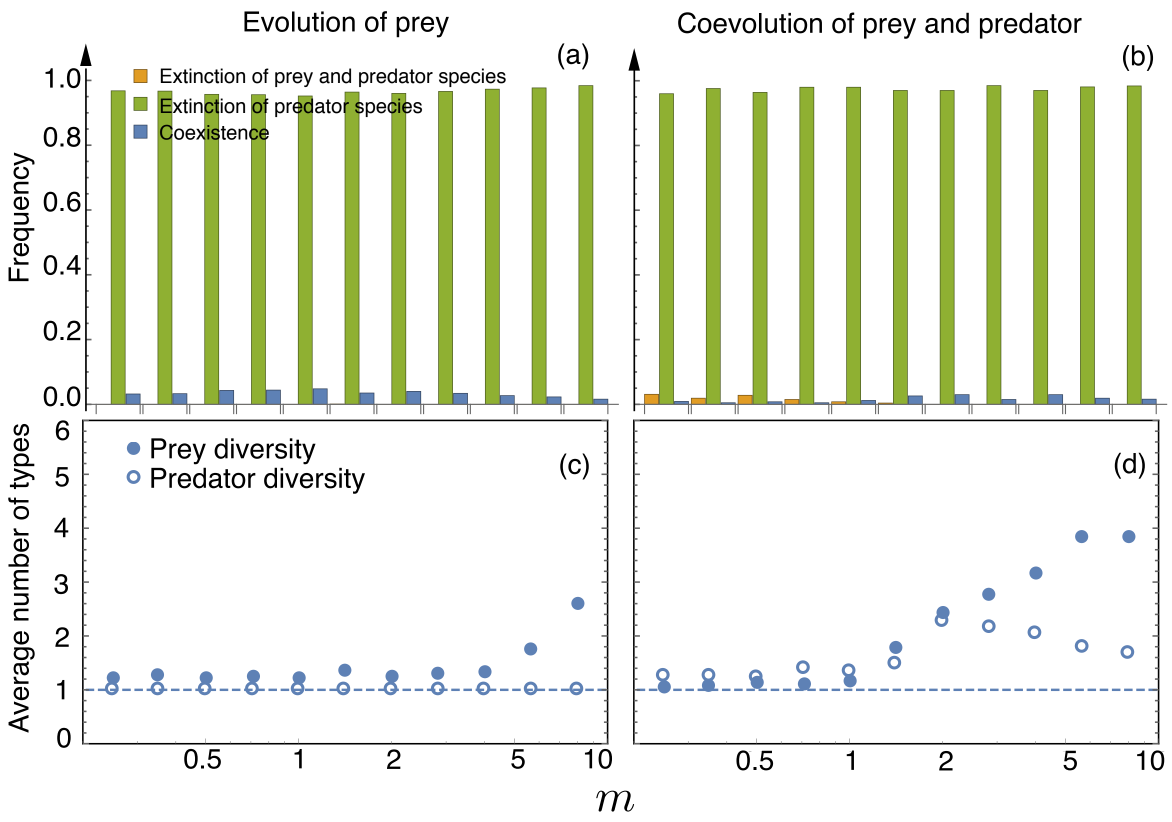

Supplementary Figure 9: **Coevolutionary and evolutionary diversity starting with evolved prey and predator types.** The extinction of the predator species is very extremely high. If both species coevolves, their diversity can reach to similar level as the case starting with non-evolved types. If only the prey can evolve, the prey is always under a high predation pressure and can only increases its diversity when the defence is very cheap to evolve. (Other parameters: $b_x = 1.0$, $d_x = 0.1$, $\mu_x = 0.0001$, $r_c = 0.00005$, $d_y = 0.5$, $\mu_y = 0.001$, $p = 0.005$, $X_1(0) = 1000$, $Y_1(0) = 100$, $g_1 = 0.2$, $k_1 = 0.06$, averaged over 1000 independent runs and time period equals to 2000 per each run. The values for parameter m in the upper panels correspond to the points in the lower panels).

Supplementary Note 8: The Shannon index

We measured the species diversity by the average number of prey and predator types. Here, we further explore whether this measurement reveals a diversity pattern consistent with a Shannon index. The Shannon index (or entropy) is defined as $-\sum_i p_i \ln p_i$, where p_i is the frequency of any present type i. Thus, not only the number of types but also their frequencies are counted.

We observe the same diversity pattern in the average number of types (Fig. 5c&d in the main text) and the Shannon index (Supplementary Fig. 10). When $m < 1$, the prey and predator diversity is low under both evolution of the prey and the coevolution prey and predator. When $m > 1$, the prey diversity increases with m and is higher under the evolutionary than the coevolutionary process. Under the coevolution scenario, the predator diversity increases first and decreases again with m.

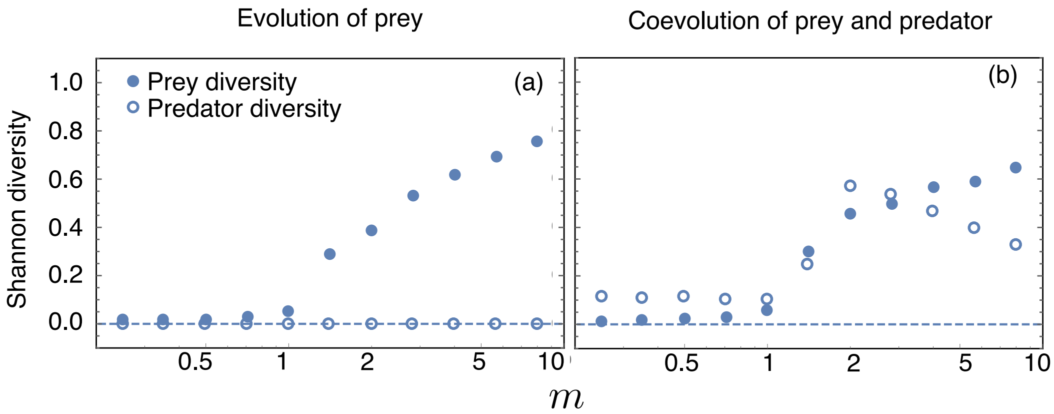

Supplementary Figure 10: **The Shannon diversity under the evolutionary and coevolutionary processes.** The diversity pattern and differences between the two processes consist with the diversity measured by the average number of types (parameters: all data points are obtained from the same simulations as in the Fig. 5 of the main text).

Supplementary Note 9: Diminishing returns in prey growth in the absence of predators

We observe the extinction of the predator species in approximately 20% of our stochastic simulations when $m < 1$ and around 5% when $m > 1$. This often happens when a prey type with extremely high defence level arises and fixes by chance (Supplementary Fig. 11b). The predation rate of non-adapted ancestor predator type decreases significantly and can lead to a sudden decrease in predator population size and even extinction due to the high stochasticity in small populations (Supplementary Fig. 11d). Once the predation pressure disappears, there is only one single fitness optimum for

the prey species, i.e., a minimal defence and maximal growth rate $g_i = 1$. In such a situation, the change of the prey growth rate towards the maximum is not continuous, but occurs in a certain number of jumps depending on each stochastic realisations. The initial jumps are likely large and then the changes become smaller when the prey growth rate approaches the optimum.

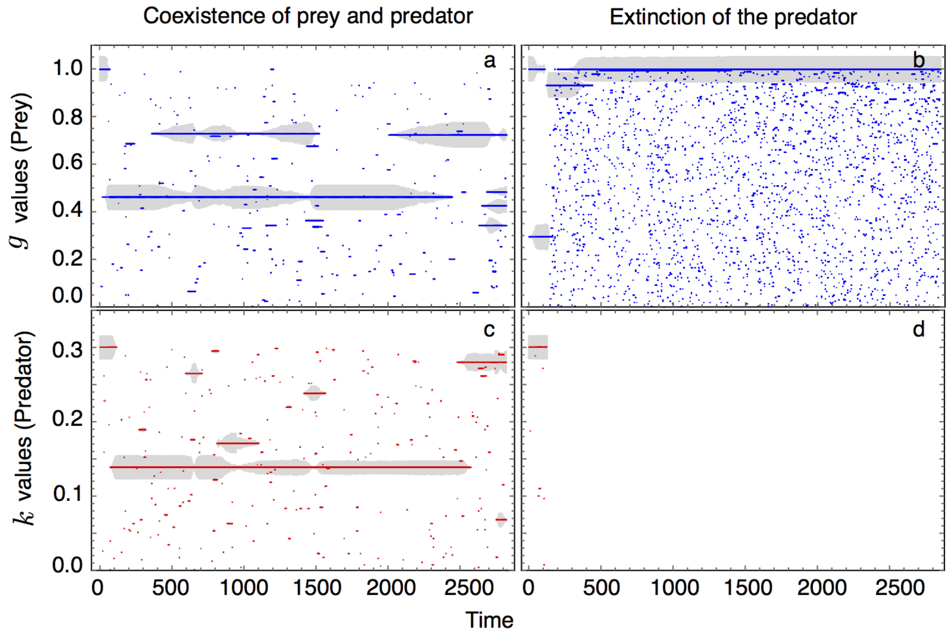

Supplementary Figure 11: **Coexistence vs. extinction of the predator under co-evolution.** Left panels: Prey and predator coexist and diversity is maintained. Right panels: the ancestor predator type goes extinct (panel d) stochastically due to the prevalence of a highly defended prey type (panel b between time 10 to 120). The extinction of the predator species leads to diminishing returns of the prey types with high growth but low defence. The width of the grey area represents the frequency of the corresponding type. The maximum frequency 1.0 has a width of 0.1 as the starting types (parameter set: $b_x = 1.0$, $d_x = 0.1$, $\mu_x = 0.0001$, $r_c = 0.00005$, $d_y = 0.5$, $\mu_y = 0.001$, $p = 0.005$, $m = 3$, $X_1(0) = 1000$, $Y_1(0) = 100$, $g_1 = 1.0$, $k_1 = k_{\text{max}} = 0.3$).

Also in Supplementary Fig. 11, we can see that the rate of evolution changes over time. Here, we refer to the rate of evolution as the emergence rate of novel types within the population, i.e., the mutation supply per time. If the prey types evolve higher defence capabilities, their reproduction rates decrease, which then decreases the number of mutations per unit time, see Supplementary Fig. 11c between time steps 200 to 400. Although the predator went extinct and selection favours the lowest defence level, the

prey population remains for some time at the high defence level. This is due to the low supply of new mutations and prey types. It takes time until *de novo* mutations of lower defence levels and thus higher growth rates appear. Once such a type occurs, it spreads fast and with the higher growth rates and increasing population sizes, the number of mutations per unit time increases significantly, see Supplementary Fig. 11c after time 400. Consequently, the prey population moves faster, but in smaller steps towards its fitness peak.

The link between the level of defence of the prevalent prey types and the rate of evolution provides an interesting and novel mechanism that stabilise predator-prey cycles with stochastic demographic fluctuations. As we show above, high defence is related to a low rate of evolution as growth rates are low. Similar to the scenario where the predator went extinct, the predation pressure is negligible when the predator density is very low. If a highly defended prey population would evolve low defence levels very rapidly, the predator population — which has coevolved with the highly defended prey population — could respond to the increase of available prey individuals rapidly and resulting in high predator population sizes. Consequently, this will decrease the prey density to a low level and even result in the extinction of the prey followed by the extinction of the predator. In this sense, the link between the rate of evolution and the present genotypes introduces a natural delay, which helps both the prey and predator populations to avoid extremely large fluctuations and potentially lowers the risk of extinction.
