# Supplementary material for: Dynamical trade-offs arise from antagonistic coevolution and decrease intraspecific diversity
Source: Nat Commun. 2017 Dec 12;8:2059. doi: 10.1038/s41467-017-01957-8 (PMC5727225; doi:10.1038/s41467-017-01957-8)
Supplement: Supplementary file 3 — Description of Additional Supplementary Files [file 41467_2017_1957_MOESM3_ESM.pdf]

File Name: Supplementary Data 1
Description: Growth rates of cilliate and bacteria in each combination of prey and predator populations
